# B-vitamins and body composition: integrating observational and experimental evidence from the B-PROOF study

**DOI:** 10.1007/s00394-019-01985-8

**Published:** 2019-05-10

**Authors:** Sadaf Oliai Araghi, Kim V. E. Braun, Nathalie van der Velde, Suzanne C. van Dijk, Natasja M. van Schoor, M. Carola Zillikens, Lisette C. P. G. M. de Groot, Andre G. Uitterlinden, Bruno H. Stricker, Trudy Voortman, Jessica C. Kiefte-de Jong

**Affiliations:** 1 Department of Internal Medicine, Erasmus University Medical Center, Postbus 2040, 3000 CA Rotterdam, The Netherlands; 2 Department of Epidemiology, Erasmus University Medical Center, Postbus 2040, 3000 CA Rotterdam, The Netherlands; 3 Department of Geriatric Medicine, Franciscus Gasthuis & Vlietland, Postbus 215, 3100 AE Schiedam, The Netherlands; 4 Section of Geriatric Medicine, Department of Internal Medicine, Academic Medical Centre, Amsterdam Public Health Research Institute, Postbus 22660, 1100 DD Amsterdam-Zuidoost, The Netherlands; 5 Department of Epidemiology and Biostatistics, Amsterdam Public Health Research Institute, VU University Medical Center, Postbus 7057, 1007 MB Amsterdam, The Netherlands; 6 Division of Human Nutrition and Health, Wageningen University, Postbus 9101, 6700 HB Wageningen, The Netherlands; 7 Department of Public Health and Primary Care, Leiden University Medical Center/LUMC Campus, The Hague, The Netherlands

**Keywords:** Vitamin B12 and folic acid, Body composition, BMI, Fat (Free) mass, Effect of vitamin B12 and folic acid on obesity

## Abstract

**Purpose:**

Higher folate and vitamin-B12 have been linked to lower risk of overweight. However, whether this is a causal effect of these B-vitamins on obesity risk remains unclear and evidence in older individuals is scarce. This study aimed to assess the role of B-vitamin supplementation and levels on body composition in older individuals.

**Methods:**

A double-blind, randomized controlled trial in 2919 participants aged ≥ 65 years with elevated homocysteine levels. The intervention comprised a 2-year supplementation with a combination of folic acid (400 µg) and vitamin B12 (500 µg), or with placebo. Serum folate, vitamin-B12, active vitamin-B12 (HoloTC), methylmalonic acid (MMA), and anthropometrics were measured at baseline and after 2 years of follow-up. Dietary intake of folate and vitamin-B12 was measured at baseline in a subsample (*n* = 603) using a validated food-frequency questionnaire. Fat mass index (FMI) and fat-free mass index (FFMI) were assessed with Dual Energy X-ray absorptiometry (DXA).

**Results:**

Cross-sectional analyses showed that a 1 nmol/L higher serum folate was associated with a 0.021 kg/m^2^ lower BMI (95% CI − 0.039; − 0.004). Higher HoloTC (per pmol/L log-transformed) was associated with a 0.955 kg/m^2^ higher FMI (95% CI 0.262; 1.647), and higher MMA (per μgmol/L) was associated with a 1.108 kg/m^2^ lower FMI (95% CI − 1.899; − 0.316). However, random allocation of B-vitamins did not have a significant effect on changes in BMI, FMI or FFMI during 2 years of intervention.

**Conclusions:**

Although observational data suggested that folate and vitamin B12 status are associated with body composition, random allocation of a supplement with both B-vitamins combined versus placebo did not confirm an effect on BMI or body composition.

**Electronic supplementary material:**

The online version of this article (10.1007/s00394-019-01985-8) contains supplementary material, which is available to authorized users.

## Introduction

The prevalence of overweight is growing in all age categories [1]. Among people older than 65 year in the European Union the prevalence of overweight, defined as a BMI of ≥ 25, has been estimated at between 58 and 66% [2]. Overweight increases the risk of several chronic diseases, including cardiovascular diseases, metabolic syndrome, and type 2 diabetes [3]. For adequate prevention of these diseases, it is, therefore, important to identify factors that influence the development of overweight across age categories.

Several studies showed that there is an association of overweight and obesity with lower serum vitamin-B12 and folate levels [4–8]. However, it is currently not clear whether deficiencies of these B vitamins are a cause or a consequence of obesity [9]. It is suggested that being overweight or obese can alter the absorption, distribution, metabolism and/or excretion of micronutrients, which may cause vitamin deficiencies, including those of folate and vitamin-B12 [10–12]. This may be particularly of importance in older persons, as this group is at higher risk for deficiencies [13], including vitamin-B12 deficiency [14], due to inadequate dietary intake as well as malabsorption and higher depletion [15]. Another explanation of the observed associations could be that B-vitamin deficiencies may contribute to adiposity, for example by the role of folate and vitamin-B12 in epigenetics. Vitamin-B12 and folate act as co-factors in one-carbon metabolism, which is important for the production of methyl donors for DNA methylation [16, 17]. Deficiency of these nutrients could lead to dysregulation of DNA methylation and might generate metabolic disturbances, including disturbed energy and lipid metabolism, contributing to adiposity [18, 19].

To date, studies exploring the association between folate and vitamin-B12 with body composition in older individuals are scarce. Furthermore, BMI as a measure of overweight in elderly population is still under debate [20]. As aging is associated with loss of muscle mass [21], BMI could underestimate the prevalence of obesity in this population. Hence, it is important to study more comprehensive measures of body composition in older people, e.g., by making a distinction between fat mass and fat free mass.

We aimed to: (1) assess the cross-sectional associations of serum folate and vitamin-B12 levels as well as dietary intake of folic acid and vitamin-B12 from both food and supplements with body composition in older individuals; and (2) to study the causal effect of these vitamins by assessing the effect of supplementation with folic acid and vitamin-B12 combined on body composition in a randomized controlled trial.

## Methods

### Study design and participants

For the present study, baseline data (2008–2011) and 2-year follow-up (2010–2013) data from the B-PROOF study (B-vitamins for the prevention of osteoporotic fractures) were used. The B-PROOF study is a multi-center, randomized, placebo-controlled, double-blind intervention study, investigating the effect of a 2-year daily oral vitamin-B12 (500 µg) and folic acid (400 µg) supplementation on fracture incidence. The study was conducted by three research centers in the Netherlands: VU University Medical Center (Amsterdam), Wageningen University (Wageningen), and Erasmus University Medical Center (Rotterdam). This study included 2919 individuals, aged 65 years and older with elevated homocysteine levels (12–50 µmol/L). Participants were excluded if they had renal insufficiency (creatinine level > 150 µmol/L) or diagnosed with malignant cancer in the past 5 years. A detailed description of the trial has been reported elsewhere [22]. All participants gave written informed consent before the start of the study. The B-PROOF study has been registered in the Netherlands Trial Register (NTRNTR1333) and with ClinicalTrials.gov (NCT00696514). The Wageningen University Medical Ethics Committee approved the study protocol, and the Medical Ethics committees of Erasmus MC and VUmc gave approval for local feasibility [22].

### Anthropometrics measurements

At baseline and follow-up, height was measured in duplicate to the nearest 0.1 cm with the participant standing erect and without wearing shoes, using a stadiometer [22]. Weight was measured to the nearest 0.5 kg using a calibrated weighing device (SECA 761) with the participant wearing light garments, empty pockets and without wearing shoes [22]. BMI was calculated as weight in kilograms divided by square of height in meters and expressed as kg/m^2^. Participants were categorized in underweight (BMI < 20 kg/m^2^), normal weight (BMI 20 to < 25 kg/m^2^), overweight (BMI 25 to < 30 kg/m^2^), or obese (BMI ≥ 30 kg/m^2^) [23].

### Body composition measurements

At baseline and follow-up, a subsample of participants from the Amsterdam and Rotterdam research centers (*n* = 424 and *n* = 803 at baseline; *n* = 380 and *n* = 732 at follow-up, respectively) underwent Dual Energy X-ray assessment (DXA) using the GE Lunar Prodigy device (GE Healthcare, USA, CV = 0.08%) (Erasmus MC) or the Hologic QDR 4500 Delphi device (Hologic Inc., USA, CV = 0.45%) (VuMC). The two devices were cross-calibrated by measuring a European spine phantom (ESP) five times on both devices and all results were adjusted accordingly. Total fat mass and total fat free mass were estimated from the DXA scan [22]. Fat Mass Index (FMI) and Fat Free Mass Index (FFMI) were calculated as total fat mass or total fat free mass in kilograms divided by square of height in meters and expressed as kg/m^2^. Android/gynoid fat ratio was calculated as fat mass android region (g)/fat mass gynoid region (g) and was only available for the participants from Rotterdam (*n* = 800).

### Laboratory measurements

Venous blood samples were obtained in the morning at baseline, when the participants were in a fasted state, or had taken a restricted breakfast. Plasma homocysteine was determined at baseline, using the Architect i2000 RS analyser (VUmc, intra assay CV = 2%, inter assay CV = 4%), HPLC method [22] (WU, intra assay CV = 3.1%, inter assay CV = 5.9%) and LCMS/MS (EMC, CV = 3.1%). According to a cross-calibration, different methods of determined plasma homocysteine of the three centers did not differ significantly. Serum folate was determined by immunoelectrochemiluminescence on a Roche Modular E170 (Roche, Almere, The Netherlands) (CV = 5.9% at 5.7 nmol/L and 2.8% at 23.4 nmol/L). Serum methylmalonic acid (MMA) was measured by LC–MS/MS (CV < 9%) and holo-transcobalamin (HoloTC), and was determined by the AxSYM analyser (Abbott Diagnostics, Hoofddorp, the Netherlands) (CV < 8%) [22, 25]. HoloTC was used as measure of vitamin-B12 status, because it has been shown to better reflect vitamin-B12 status than serum total vitamin-B12 [26]. To isolate DNA, buffy coats were used. The MTHFR genotypes, 677CC, 677CT or 677 TT, were determined using the Illumina Omni-express array (Illumina Inc., San Diego, CA, USA).

### Food intake measurements

Dietary intake was estimated at baseline in a subsample, i.e., all participants of the Wageningen region (*n* = 603), by a 190 item Food Frequency Questionnaire (FFQ), originally designed to assess intake of energy, total fat, fatty acids and cholesterol. This FFQ was updated using the Dutch National Food Consumption Survey of 1998 and extended with questions to estimate other macronutrients, vitamin-B12, folate, vitamin D, and calcium intake by the dietetics group of Wageningen University [27, 28]. When one item contributed to more than 0.1% of the intake of macronutrients, vitamin-B12, folate, vitamin D or calcium in this national survey, this item was added to the FFQ. In the end, all items in the FFQ accounted for 90% of the total folate and vitamin-B12 intake according to Dutch National Food Consumption Survey data of 1998. The Dutch Food Composition database (NEVO) was used to calculate daily folate and vitamin-B12 intake [29].

All dietary nutrient intake were adjusted for total energy intake using the residual method [30]. The participants were also asked to write down the brand names of each supplement they used in the questionnaire at the baseline. Current use of folic acid and/or vitamin-B12 supplement was defined as users or non-users [22]. Total amount of folic acid and vitamin-B12 supplement was also measured from the FFQ.

### Covariates

Demographic characteristics and health status, which included age, sex, self-reported medical history (cardiometabolic diseases, i.e., cardiovascular disease, diabetes, hypertension and hypercholesterolemia), alcohol intake, smoking habits, physical activity (PA, from the LASA Physical Activity Questionnaire, total activity expressed in kcal/day), education, were determined using a structured questionnaire. Alcohol intake was categorized into ‘never’, ‘light’, ‘moderate’ and ‘excessive’, based on the number of days per week alcohol was consumed and the number of glasses per time, following the Dutch method of Garretsen et al. [31, 32] smoking habits were defined as never smoked; former smoker, or current smoker.

### Statistical analysis

Normal distribution for all variables was examined by visual inspection of histograms. When necessary, data were log-transformed. For the cross-sectional analyses at baseline, linear regression analysis was used to determine associations of serum folate, vitamin-B12, HoloTC, MMA, folic acid intake, vitamin-B12 intake, folic acid supplement use, and vitamin-B12 supplement use, and total folic acid and vitamin B-12 (from food and supplement) with BMI, FMI and FFMI. All analyses were adjusted for age and sex, and for energy intake for the associations for folic acid and vitamin-B12 intake (from food and supplements) (model 1). Subsequently, based on literature, smoking, alcohol, PA, education, hypertension, and hypercholesterolemia were added as confounders [33, 34]. Furthermore, low serum vitamin-B12 and folate levels are characteristics of overweight and obese subjects, therefore, we stratified the population into three different groups, normal weight (BMI < 25 kg/m^2^), overweight (BMI 25-30 kg/m^2^) and obesity (BMI > 30 kg/m^2^).

For the analyses on the effect of the intervention, the primary analyses were performed according to the intention-to-treat (ITT) principle. To study the effect of the treatment on BMI, FMI and FFMI, linear regression analyses were performed for the subjects with at least one observation of height and weight (baseline and/or follow-up). The independent variable as randomization group (intervention vs. placebo) and the dependent variables were measures of body composition at follow-up as well as the difference between baseline and follow-up measurement (delta BMI, delta FMI and delta FFMI).The effect of the treatment on delta body composition was adjusted for baseline measurement of body composition and HoloTC (because significant differences in HoloTC was present between the treatment and control group).

In addition, for the experimental analyses, we assessed whether our findings were different by age, gender, homocysteine concentrations, presence of cardiometabolic disease, and genetic vulnerability, by testing the interaction of these potential effect modifiers with the intervention. If the P value for interaction was < 0.1, stratified analyses were performed. Statistical analyses were performed using the statistical software package of SPSS 24.0 (SPSS Inc., Chicago, Illinois, USA). *P* values of < 0.05 were considered statistically significant for all the analyses other than the interaction analyses (< 0.1).

## Results

### Population characteristics

Population baseline characteristics are presented in Table 1. Mean age was 74.0 years (SD 6.5) for the total population (*n* = 2919), 72.9 years (SD 5.7) for the DXA population (*n* = 1227) and 72.8 years (SD 5.7) for the population with dietary intake data (*n* = 603). In the total population, mean BMI at baseline was 27.1 kg/m^2^ for the intervention group and 27.2 kg/m^2^ and control group. In the DXA population, mean FMI and FFMI were 8.9 and 18.0 kg/m^2^, respectively, for the intervention group and 8.9 and 18.1 kg/m^2^, respectively, for the control group at baseline. Mean baseline folic acid level of total population was 21.0 nmol/L (SD 11.62) and mean baseline vitamin-B12 level was 285.5 pmol/L (SD 116.0). Follow-up measurements were conducted 2 years after in 2636 participants. The characteristics of these participants are shown in Supplemental Table 1. Mean folic acid level of total population raised to 40.5 nmol/L and for vitamin-B12 level the mean raised to 472.6 pmol/L (SD 378.2) after the intervention.

**Table 1:** Population baseline characteristics

	B-PROOF participants (*N* = 2919)	DXA-test participants (*N* = 1227)	FFQ participants (*N* = 603)
Age (years)^a^	74 (6.5)	72.9 (5.7)	72.8 (5.7)
Sex			
Female (%)	50	48.3	42.1
Body mass index (kg/m^2^)^a^	27.1 (4.0)	27.0 (3.8)	26.9 (3.6)
Underweight (%)	0.4	0.2	0.3
Normal weight (%)	28.6	30.4	28.4
Overweight (%)	50.9	50.1	54.7
Overweight (%)	20.1	19.2	16.6
Fat	NA		NA
Total fat mass (kg)		25.5 (8.4)	
Total fat percentage (%)		32.4	
FMI (kg/m^2^)		8.9 (3.2)	
FFMI (kg/m^2^)		18.0 (2.2)	
Smoking (%)			
Current	56.5	57.6	58.8
Former	9.6	9.0	10.4
Never	33.9	33.3	31.0
Alcohol intake (%)			
Light	67.4	64.1	64.2
Moderate	28.8	31.4	32.8
Excessive	3.4	4.0	2.5
Very excessive	0.4	0.6	0.5
Self-reported medical history of			
Cardiac disease (% yes)	25.1	25	25.5
Diabetes (% yes)	10.3	10.8	7.1
Hypercholesterolemia (%yes)	24.7	28.5	21.2
Measured hypertension (%yes)	51.5	58.8	38.6
Homocysteine (mmol/L)^b^	14.4 [3.4]	14.3 [3.2]	14.0 [3.2]
Serum folate (nmol/L)^a^	21.0 (11.62)	21.3 (9.3)	20.1 (17.2)
Serum vitamin-B12 (pmol/L)^a^	285.5 (116.0)	287.5 (115.2)	281.2 (107.9)
Holotranscobalamin (pmol/L)^b^	64.0 [251.0]	67.0 [41.0]	60.0 [34.0]
MMA (µgmol/L)^b^	0.2 [0.1]	0.2 [0.1]	0.2 [0.1]
MTHFR (%)			
CC	44.9	46.4	44.3
CT	42.1	40.8	42.6
TT	13.0	12.8	13.1
Folic acid supplement use (%)	16.0	16.3	11.1
Vitamin-B12 supplement use (%)	16.2	16.8	11.1
Folate intake from food (mcg/day)^a^	NA	NA	191.5 (53.9)
Vitamin-B12 intake from food (mcg/day)^a^	NA	NA	4.1 (2.0)
Total activity (Kcal/day)^b^	560 [489]	595 [504]	593 [506]
Total energy intake (Kcal/day)^a^	NA	NA	2006 (473)
Education (%)			
Low	32.1	32.5	25.9
Middle	42.0	41.1	40.8
High	26.0	26.4	33.3
Region (%)			
Amsterdam	26.6	34.6	0
Rotterdam	29.4	65.4	0
Wageningen	44.0	0	100

^a^Presented as mean (SD)^b^ median [IQR]

### Baseline associations of vitamin B12 and folic acid with body composition

Results of the cross-sectional linear regression analyses of serum folate, serum vitamin-B12, HoloTC, MMA, folic acid supplement use, and vitamin-B12 supplement use with BMI for the total population, are shown in Table 2. Higher serum folate (per nmol/L) was associated with a 0.021 kg/m^2^ lower BMI (95% CI − 0.039; − 0.004) after adjustments for covariates. Serum vitamin-B12, HoloTC, MMA, folic acid supplements and vitamin-B12 supplements were not associated with BMI after adjustment for covariates (Table 2). We found also no significant associations between intake of folic acid and vitamin-B12 (total and from food only) and BMI (Table 2).

**Table 2:** Baseline associations of serum folate, vitamin-B12, HoloTC, MMA, folic acid supplements and vitamin-B12 supplements with BMI

	BMI	
	Model 1	Model 2
	*β* 95% CI	*β* 95% CI
Serum folate (nmol/L) (*n* = 2919)	− 0.018 [− 0.030; − 0.005]*	− 0.021 [− 0.039; − 0.004]*
Serum vitamin-B12 (pmol/L) (*n* = 2919)	− 0.001 [− 0.003; − 0.00001]*	− 0.001 [− 0.002; 0.001]
HoloTC_log^a^ (pmol/L) (*n* = 2919)	0.533 [− 0.085; 1.151]	0.612 [− 0.099; 1.323]
MMA (μgmol/L) (*n* = 2919)	− 0.258 [− 0.742; 0.225]	− 0.675 [− 1.470; 0.120]
Folic acid supplement use (*n* = 2919)	− 0.271 [− 0.666; 0.124]	− 0.104 [− 0.543; 0.335]
Vitamin-B12 supplement use (*n* = 2919)	− 0.366 [− 0.758; 0.026]	− 0.268 [− 0.705; 0.169]
Folic acid total intake (FFQ) (*n* = 1227)	− 0.0002 [− 0.002; 0.001]	0.0005 [− 0.003; 0.002]
Vitamin-B12 total intake (FFQ)^a^ (*n* = 1227)	− 0.795 [− 2.086; 0.496]	− 1.045 [− 2.735; 0.646]
Folic acid intake from food (FFQ) (*n* = 1227)	0.005 [− 0.002; 0.011]	0.007 [− 0.001; 0.015]
Vitamin-B12 total intake from food (FFQ) (*n* = 1227)	0.033 [− 0.013; 0.080]	0.069 [− 0.006; 0.143]

Values are regression coefficients and 95% CIs based on linear regression models and reflect differences in BMI per 1 unit increase of serum folate, serum vitamin-B12, HoloTC, MMA, folic acid supplements, vitamin-B12 supplements

Model 1 is adjusted for age and sex

Model 2 is additionally adjusted for smoking, alcohol consumption, physical activity, education, hypertension and hypercholesterolemia

^a^log transformed

*P value < 0.05

Results in a subgroup (*n* = 1227) with DXA measurements showed that there was an association between HoloTC and FMI (*β* 0.955 kg/m^2^ per pmol/L; 95% CI 0.262; 1.647) in the adjusted model, indicating that for each pmol/L higher log-transformed HoloTC, there was a 0.955 kg/m^2^ higher FMI. MMA was significantly inversely associated with FMI, indicating that for each μgmol/L higher MMA, there was a 1.108 kg/m^2^ lower FMI (95% CI − 1.899; − 0.316). None of the examined exposures were associated with FFMI (Table 3) or with android/gynoid fat ratio (data not shown).

**Table 3:** Baseline associations of serum folate, vitamin-B12, HoloTC, MMA, folic acid supplements and vitamin-B12 supplements with FMI and FFMI in the population with DXA data (*n* = 1227)

	FMI		FFMI	
	Model 1	Model 2	Model 1	Model 2
	*β* 95% CI	*β* 95% CI	*β* 95% CI	*β* 95% CI
Serum folate (nmol/L)	− 0.002 [− 0.019; 0.016]	− 0.002 [-0.019; 0.016]	− 0.009 [− 0.020; 0.002]	− 0.010 [− 0.021; 0.0005]
Serum vitamin-B12 (pmol/L)	0.0002 [− 0.002; 0.001]	− 0.000003 [− 0.001; 0.001]	0.0001 [− 0.001; 0.001]	0.0003 [− 0.001; 0.001]
HoloTC_log^a^ (pmol/L)	0.748 [0.073; 1.423]*	0.955 [0.262; 1.647]*	0.232 [− 0.191; 0.654]	0.403 [− 0.032; 0.839]
MMA (μgmol/L)	− 0.746 [− 1.530; 0.039]	− 1.108 [− 1.899; − 0.316]*	− 0.138 [− 0.629; 0.353]	− 0.170 [− 0.669; 0.328]
Folic acid supplements	− 0.031 [− 0.462; 0.400]	0.174 [− 0.272; 0.621]	− 0.175 [− 0.444; 0.094]	− 0.105 [− 0.384; 0.174]
Vitamin-B12 supplements	− 0.184 [− 0.610; 0.242]	− 0.054 [− 0.494; 0.386]	− 0.191 [− 0.457; 0.075]	− 0.112 [− 0.388;0.163]

Values are regression coefficients and 95% CIs based on linear regression models and reflect differences in BMI per 1 unit increase of serum foliate, serum vitamin-B12, HoloTC, MMA, folic acid supplements, vitamin-B12 supplements

Model 1 is adjusted for age and sex

Model 2 is additionally adjusted for smoking, alcohol consumption, physical activity, education, hypertension, hypercholesterolemia

^a^log transformed

*P value < 0.05

Stratified analysis of different groups of BMI showed that higher serum folate level was associated with a lower BMI in the population with overweight and obesity; however, the associations were not statistically significant (Supplemental Table 2).

### The effect of the intervention

After 2 years follow-up, mean BMI was 27.2 kg/m^2^ for both groups, mean FMI and FFMI were 9.1 and 18.0 kg/m^2^, respectively, for the intervention group, and 9.0 and 18.0 kg/m^2^, respectively, for the control group. Linear regression analyses showed that the combined vitamin B12 and folic acid intervention did not affect changes in BMI (*β* = − 0.051; 95% CI − 0.368; 0.265 for the effect on FU BMI and *β* = − 0.031; 95% CI − 0.156; 0.093 for the effect on delta BMI in the total population) (Table 4). Additionally, no significant effect of the intervention on changes in FMI or FFMI was observed (Table 4).

**Table 4:** The effect of the intervention on follow-up and changes of body composition

	Intervention group			Placebo group				
	Baseline estimated mean (SD)	FU estimated mean (SD)	2-year change	Baseline estimated mean (SD)	FU estimated mean (SD)	2-year change	Treatment effect on FU *β* 95% CI	Treatment effect on ∆ BC *β* 95% CI
BMI (*n* = 2919)	27.116 (3.975)	27.158 (4.229)	0.042	27.171 (3.959)	27.207 (4.008)	0.049	− 0.051 [− 0.368; 0.265]	− 0.031 [− 0.156; 0.093]
FMI (*n* = 1227)	8.951 (3.201)	9.120 (3.244)	0.169	8.929 (3.227)	8.975 (3.110)	0.046	0.105 [− 0.270; 0.479]	0.015 [− 0.101; 0.130]
FFMI (*n* = 1227)	17.979 (2.150)	17.976 (2.182)	− 0.003	18.057 (2.273)	17.951 (2.172)	− 0.106	0.039 [− 0.220; 0.298]	0.052 [− 0.073; 0.177]

Values are regression coefficients and 95% CIs based on linear regression models and reflect differences in BMI, FMI, and FFMI for intervention compared to the placebo group

*FU* follow-up, *∆ BC* difference between body composition at baseline and follow-up

### Additional analyses on the intervention

For the experimental analyses, we observed a significant interaction between treatment and gender with BMI (*p* for interaction 0.001), cardiometabolic diseases with FMI and FFMI at the end of follow-up (*p* for interaction 0.06 and 0.02 resp.), and MTHFR with change in FFMI (*p* for interaction 0.05). Stratified analyses showed only a significant effect of the intervention for people with MTHFR genotype TT, compared to CT and CC (*β* 0.46, 95% CI 0.13; 0.79 and *β* − 0.05, 95% CI − 0.26; 0.16 and *β* − 0.04, 95% CI − 0.25; 0.18 resp.) (Supplemental Table 3).

## Discussion

In the current study, we integrated observational and experimental data on B-vitamins and body composition in a large elderly population. Although we found that higher serum folate was associated with a lower BMI and, that indices of a higher vitamin-B12 status were associated with a higher FMI, we did not observe any effect of random allocation with both B-vitamins combined on BMI or body composition after 2 years of intervention.

Hypotheses of the observed associations between B-vitamins and body composition have been proposed for both directions: folate and vitamin-B12 deficiency may be a consequence of obesity due to inadequate dietary intake, altered absorption and (urinary) excretion of folate and vitamin-B12 [35], but may also cause obesity via epigenetic mechanisms, such as DNA methylation and miRNA expression involved in lipid homeostasis and inflammatory pathways [18, 19, 36]. In our observational data, we confirmed results from previous studies showing associations between B-vitamin status and body composition. However, we did not find any differences in body composition after supplementation with folic acid and vitamin-B12 after 2 years of follow-up, suggesting that B-vitamins may not have a role in the etiology of obesity or changes in body composition in the older individuals. In addition to evaluate whether insufficient dietary intake explained the results, we examined the associations between dietary intake of folate and vitamin-B12 with body composition which also did not show that (dietary intake) vitamin-B12 and folate were related to differences in body composition. Thus, on the basis of our findings, it may be argued that obesity or increased fat mass may lower B-vitamins status [37], but not the other way around. This is in line with a previous study in which the causality of the relation between vitamin-B12 and BMI was studied with use of a Mendelian randomization approach. The authors reported that vitamin-B12 levels were associated with BMI, however, there was no evidence that lower vitamin-B12 levels caused a higher BMI [38]. In our observational data, we found a non-significant association of a lower BMI with higher intake of vitamin-B12. In contrast to our hypothesis, we observed that several biomarkers of higher vitamin-B12 status, including higher levels of HoloTC and lower levels of MMA, were associated with a higher FMI. In contrast, in another study among adults from a primary care-based setting vitamin-B12 level was negatively correlated with BMI [6]. With regard to folate, Kimmons et al. showed that, compared with normal-weight adults, overweight and obese adults were more likely to have low folate levels [4]. Similarly, a study by Mahabir et al. showed that adiposity was associated with lower serum folate levels in postmenopausal women [5], suggesting that obese individuals are at increased risk of folate deficiency.

We found an indication that the B-vitamin intervention had an effect on changes in FFMI in participants with the MTHFR TT variant, and not in the those with the CT and CC variant. It is known that the TT variant is associated with higher risk of folate deficiency and increased homocysteine concentrations, which may suggest that a potential effect of B-vitamins on change in fat free mass may be restricted to elderly population at risk for folate deficiency or disturbances in one-carbon metabolism, but this needs further replication in larger intervention studies.

### Strengths and limitations

The current study has several strengths and limitations. An important strength is the size of the population included and the combination of both observational and experimental data. Another strength of this study was that detailed body composition measurements were available using DXA. Although BMI may be practical to measure, it is limited regarding prediction of several health outcomes, especially in older individuals, because it does not take into account body composition [39, 40]. We had also several methods to measure vitamin-B12 levels which provided better insight into potential deficiencies. For instance, HoloTC has been shown to be a more accurate to detect vitamin-B12 deficiency compared to other methods [41]. Also serum concentrations of MMA are considered to be metabolic indicators of vitamin-B12 status through the L-Methylmalonyl-CoA mutase [42].

To appreciate our findings also some limitations should be taken into account. We used the intervention data to show that folic and vitamin-B12 supplementation did not affect body composition. Thus, our findings suggest that obesity or increased fat mass may lower B-vitamin status, but not vice versa. However, due to potential residual confounding we cannot conclude that the cross-sectional association between biomarkers of vitamin-B12 with FMI is explained by an effect of adiposity on B12 levels. Associations may also be due to, e.g., other dietary and lifestyle factors, which unfortunately we could not explore in this population. Furthermore, due to the low variation of B-vitamin status in our study population, we may not have been able to detect any possible effect of the intervention on body composition and the results are less generalizable in other populations. The combination of vitamin B12 and folic acid in the intervention may have limited our ability to detect an individual effect of these vitamins on body composition. In cross-sectional analyses, we observed associations in different directions for folate levels and biomarkers of vitamin-B12. Therefore, a potential effect of either of these vitamins on body composition could not be detected in our experimental data, as these may have canceled each other. Replication of findings forthcoming this study are thus required to examine whether results are true associations and should be examined for vitamin B12 and folic acid separately.

## Conclusion

In this large population of older individuals, although observational evidence suggested that folate and vitamin B12 are associated with body composition, random allocation of a supplement with both B-vitamins versus placebo did not confirm an effect on body composition. Future studies should further investigate mechanisms underlying potential effects of body composition on B-vitamin status and how these effects may differ for certain subgroups.

## Electronic supplementary material

Below is the link to the electronic supplementary material.

Supplementary material 1 (DOCX 26 kb)
