# Supplementary material for: B-vitamins and body composition: integrating observational and experimental evidence from the B-PROOF study
Source: Eur J Nutr. 2019 May 10;59(3):1253–62. doi: 10.1007/s00394-019-01985-8 (PMC7098930; doi:10.1007/s00394-019-01985-8)
Supplement: Supplementary file 1 — Supplementary material 1 (DOCX 26 kb) [file 394_2019_1985_MOESM1_ESM.docx]

**Supplementary data**

**Supplemental Table 1: Population follow up characteristics**

|  | **B-PROOF Participants (N =2636)** |
| --- | --- |
| Age (years)^a^ | 75.6 (6.2) |
| Sex  Female (%) | 50 |
| Body Mass Index (kg/m^2^)^a^  Underweight (%)  Normal weight (%)  Overweight (%)  Obesity (%) | 27.2 (4.1)  0.4  29.5  48.8  21.2 |
| Fat  Total Fat Mass (kg)  Total Fat Percentage (%)  FMI (kg/m^2^)  FFMI (kg/m^2^) | NA |
| Smoking (%)  Smoking not changed  Stopped smoking  Started smoking | 96.1  1.2  0.2 |
| Alcohol intake (%)  Light  Moderate  Excessive  Very excessive | 69.9  27.2  2.6  0.3 |
| Self-reported medical history of  Cardiac disease (% yes)  Diabetes (% yes)  Hypercholesterolemia (%yes)  Measured hypertension (%yes) | 16.2  11.1  25.1  56.5 |
| Homocysteine (mmol/L)^b^ | 12.2 [5.0] |
| Serum Folate (nmol/L)^a^ | 40.5 (22.6) |
| Serum Vitamin-B12 (pmol/L)^a^ | 472.6 (378.2) |
| Holotranscobalamin (pmol/L)^b^ | 87.0 [70.0] |
| MMA (µmol/L)^b^ | 0.2 [0.1] |
| MTHFR (%)  CC  CT  TT | 44.9  42.1  13.0 |
| Folic Acid supplement use (%) | 12.0 |
| Vitamin-B12 supplement use (%) | 12.1 |
| Folate intake from food (mcg/day)^a^ | NA |
| Vitamin-B12 intake from food (mcg/day)^a^ | NA |
| Education (%)  Low  Middle  High | 32.1  42.0  26.0 |
| Region (%)  Amsterdam  Rotterdam  Wageningen | 26.6  29.4  44.0 |

*^a^Presented as mean (SD)^b^ median [IQR]*

| **Supplemental Table 2: Associations between vitamin B12 and folate intake and serum and BMI - stratified for BMI (normal weight, overweight and obesity).** | | | | | | | | | | | | | |
| --- | --- | --- | --- | --- | --- | --- | --- | --- | --- | --- | --- | --- | --- |
| **BMI** | | | | | | | | | | | | |  |
|  | Model 1  β | | 95% CI | |  | | Model 2 β | | 95% CI | |  | |  |
| **Normal weight (BMI <25)** |  | |  | |  | |  | |  | |  | |  |
| Serum folate | 0.002 | -0.008 | | 0.013 | | 0.003 | | -0.010 | | 0.015 | |  |  |
| Serum vitB12 | -0.0002 | -0.001 | | 0.001 | | -0.0003 | | -0.001 | | 0.001 | |  |  |
| **Overweight (BMI 25-30)** |  |  | |  | |  | |  | |  | |  |  |
| Serum folate | -0.002 | -0.007 | | 0.003 | | -0.0003 | | -0.009 | | 0.008 | |  |  |
| Serum vitB12 | 0.0001 | -0.001 | | 0.001 | | 0.0002 | | -0.001 | | 0.001 | |  |  |
| **Obesity (BMI >30)** |  |  | |  | |  | |  | |  | |  |  |
| Serum folate | -0.024 | -0.057 | | 0.009 | | -0.013 | | -0.053 | | 0.026 | |  |  |
| Serum vitB12 | -0.0005 | -0.003 | | 0.002 | | -0.001 | | -0.004 | | 0.002 | |  |  |
|  |  |  | |  | |  | |  | |  | |  |  |

**Supplemental Table 3: The effect of the intervention on follow-up and changes of body composition stratified for effect modifiers (p-interaction < 0.10)**

|  | **Treatment effect on FU *β 95% CI*** | **Treatment effect on ∆ BC *β 95% CI*** | **Treatment effect on FU *β 95% CI*** | **Treatment effect on ∆ BC *β 95% CI*** | **Treatment effect on FU *β 95% CI*** | | **Treatment effect on ∆ BC *β 95% CI*** |
| --- | --- | --- | --- | --- | --- | --- | --- |
|  | **Without cardiometabolic diseases** | | **With cardiometabolic diseases** | |  | |  |
| **FMI** | -0.118 [-0.545; 0.309] |  | 0.661 [-0.092; 1.431] |  | **-** | | **-** |
| **FFMI** | 0.227 [-0.069; 0.522] | - | -0.432 [-0.935; 0.070] | - | - | | - |
|  | **Male** | | **Female** | |  | |  |
| **BMI** | -0.113 [-0.480; 0.254] | - | 0.011 (-0.508; 0.530) | - | - | | - |
|  | **MTHFR TT** | | **MTHFR CT** | | **MTHFR CC** | | |
| **FFMI** | - | **0.489 [0.157; 0.821]*** | - | -0.057 [-0.261; 0.147] | - | -0.032 [-0.237; 0.173] | |

*Values are regression coefficients and 95% CIs based on linear regression models and reflect differences in BMI, FMI, and FFMI for intervention compared to the placebo group.*

*FU= follow-up*

*∆ BC= difference between body composition at baseline and follow-up.*

** p-value <0.05*
